# Addressing sexual health needs in gynecological cancer survivors: insights from a tertiary cancer center in Jordan

**DOI:** 10.3389/fonc.2025.1561947

**Published:** 2025-09-30

**Authors:** Lina Wahbeh, Khawlah Ammar, Shatha Abutaha, Isam Lataifeh, Imad Jaradat, Ramiz Abu-Hijlih, Issa Mohamad, Samer Salah, Sobuh Moh’D Sobhi Abu-Shanab, Muna Al-Sayyed, Maysa Al-Husseini, Fawzi Abuhijla

**Affiliations:** ^1^ Department of Radiation Oncology, King Hussein Cancer Center, Amman, Jordan; ^2^ Research Office, King Hussein Cancer Center, Amman, Jordan; ^3^ Department of Surgery, King Hussein Cancer Center, Amman, Jordan; ^4^ Medical Oncology Department, King Fahad Specialist Hospital, Dammam, Saudi Arabia; ^5^ Psycho-social Program, King Hussein Cancer Center, Amman, Jordan; ^6^ Department of Pathology, King Hussein Cancer Center, Amman, Jordan

**Keywords:** sex education, gynecological malignancy, cancer survivor, cervical cancer, quality of life

## Abstract

**Background:**

Sexual health is a crucial aspect of quality of life, yet it is rarely addressed by healthcare providers, especially in the context of gynecological cancer. Reports on sexual education (SE) for gynecological cancer patients are particularly scarce in the Middle East and North Africa (MENA) region. This study aims to highlight the experiences, needs, and preferences of gynecological cancer survivors regarding SE during cancer treatment and follow-up.

**Methods:**

A cross-sectional survey was conducted among gynecologic cancer survivors at the Radiation Oncology Department of King Hussein Cancer Center. A data collection tool, developed and validated by a multidisciplinary panel of experts, was used to explore patient-provider discussions about sexuality and the factors influencing these discussions. These factors included both patient and healthcare provider-related aspects. Statistical analysis was performed using Chi-square and ANOVA tests.

**Results:**

Sixty married patients participated in our survey, most of whom (n=27; 45%) had cervical cancer. The mean age of patients was 52, and for their sexual partners was 57. Two-thirds (66.7%) reported sexual activity (SA) as a somewhat or very important aspect of their life. While 48% reported that their partners noted a negative impact on SA after treatment, none considered stopping treatment to preserve sexual function. Although 86% felt it was important to discuss SE at clinic, and 41.7% specified that the timing of this discussion should be before treatment, only 35% of patients had had this discussion throughout their treatment and follow up. This discussion was held by the physician in 94% of instances. The most common barrier to discussion was having a male physician (71%), followed by embarrassment (60%). In our analysis, we found that physicians tend to discuss SA with patients who had younger sexual partner’s (p value = 0.024).

**Conclusion:**

This study is the first in the MENA region to address SE in this patient population. SE is a priority for two-thirds of the patients surveyed, yet it appears that physicians are not adequately addressing this need. Further research is needed to evaluate physicians’ knowledge, attitudes, and practices regarding SE to provide comprehensive, high-quality care to gynecological cancer patients.

## Synopsis

This study investigates the often-neglected topic of sexual education (SE) for gynecological cancer survivors in the Middle East and North Africa (MENA) region, focusing on their experiences, needs, and preferences. Conducted at the King Hussein Cancer Center, the cross-sectional survey involved 60 married gynecological cancer survivors, primarily cervical cancer patients, with a mean age of 52. Two-thirds considered sexual activity important, yet only 35% had discussed SE with healthcare providers during their treatment and follow-up, mostly with male physicians, a factor identified as a key barrier (71%). Despite the importance of SE to patients, provider-patient communication was limited, influenced by embarrassment and demographic factors such as the partner’s age. The findings highlight the urgent need for improved physician training and practices to address sexual health comprehensively in this population.

## Introduction

Each year, approximately 100,000 patients in the United States are diagnosed with gynecological malignancies (1). In Jordan, the incidence of gynecological cancers in 2022 was 667 cases, with a 5-year prevalence of 2108 cases (2). Gynecological cancers and their treatments have a profound impact on sexual activity. These effects often begin with pre-diagnosis symptoms such as vulvar or pelvic pain, and or vaginal bleeding, which may or may not be triggered by intercourse. The psychological burden of a cancer diagnosis further exacerbates these issues and continues throughout the course of treatment—including surgery, chemotherapy, and radiotherapy—ultimately affecting the quality of life for survivors.

Previous studies conducted mainly outside the Middle East and North Africa (MENA) region have shown that sexual education is often inadequately addressed by healthcare providers, despite many patients’ expressing a desire for receiving related information during treatment and follow up (3, 4), another study from Jordan emphasized the lack of effective communication with physicians for sexual education (5). There is little consensus on the optimal approach and timing for delivering sexual education (6), with varying needs reported, particularly among younger patients and those in relationships (7). Gynecological cancer survivors commonly attribute their sexual dysfunction to a combination of physical (8, 9), and psycho-social concerns (10, 11). Consequently, managing sexual dysfunction following gynecological cancer therapy requires addressing both of these aspects (12, 13).

Healthcare providers should be vigilant in assessing sexual dysfunction stemming from both the disease and its treatment, applying appropriate strategies during treatment and follow-up. Delp Pub L and colleagues have outlined therapeutic strategies and recommendations tailored to the symptoms reported by survivor patients (14). Correia et al. reported that 32% of patients remained sexually active following cervical cancer treatment, though more than half of these patients experienced sexual dysfunction, as measured by the Female Sexual Function Index (FSFI) (15). Similarly, Fischer et al. found that women who survived ovarian cancer often experienced a poorer quality of sexual life (16).

Recognizing the limited reporting on this topic from the MENA region, particularly within the Arab world, our study aims to explore the sexual health education needs of our patients, focusing on the appropriate extent and timing of this education. Additionally, we seek to assess the burden of radiation-induced side effects among our patients and their impact on sexual activity.

## Methodology

This cross-sectional study included adult gynecologic cancer survivors treated at the Radiation Oncology Department of King Hussein Cancer Center (KHCC) between 2014 and 2023. Eligible participants were those who had achieved complete remission for at least six months, were married at the time of data collection, and were at least 18 years old, ovarian cancer patients were excluded because radiation treatment is not an essential part of their treatment. Eligible patients were identified through a review of approximately 250 medical charts. Many patients were excluded from the study due to being single, divorced, or widowed. All eligible patients within the six-month timeframe were approached during follow-up clinic visits and invited to complete a questionnaire. We explained the purpose, importance, and implications of the research to each patient approached for the questionnaire, reassuring them that their responses would remain anonymous and confidential. The questionnaire was developed by the research team based on several previous studies (7, 17). Its content validity was evaluated by a multidisciplinary panel of seven experts including: two gyn-oncologist, one nurse, one psychosocial expert, one medical oncologist and two radiation oncologists.

The questionnaire was designed and prepared in Arabic, with all words and phrases carefully reviewed to ensure cultural appropriateness. It included a demographic section, along with questions exploring current sexual activity and the importance of sexual activity to the patient. The final part of the questionnaire focused on patient-provider discussions about sexual activity and the factors that might influence these discussions, whether related to the healthcare provider or the patients themselves. After finalizing the questions and receiving provisional approval, we conducted a pilot phase involving 10 patients, during which no changes were necessary.

To facilitate responses, a scale was incorporated into some questions, particularly those assessing the quality of sexual life before diagnosis, the quality of sexual life after treatment, and the current importance of sexual life to the patient. This scale ranged from 1 to 10, and responses were analyzed using descriptive statistics (Mean; SD).Adapted table from Krejcie and Morgan was considered to determine minimum needed sample, and based on a p value of 0.05 and time frame for data collection, sample size of at least 55 patients is considered to be representative (18). SPSS software was used to analyze data, descriptive statistics were done, associations were measured either by using Chi-square or One-Way ANOVA tests P <0.05 considered significant.

The study was reviewed and approved by the institutional review board, approval number (22 KHCC 168).

## Results

### Patient’s demographics

Sixty patients completed the questionnaire, yielding a response rate of 95%, with only three patients declining to participate. The mean age of the participants was 52 ± 8.52 years, while the mean age of their husbands was 57 ± 10.24 years. The majority of patients were diagnosed with cervical cancer (45%, n=27), followed by endometrial cancer (31%, n=19), vulvar cancer (20%, n=12), and other types (3.3%, n=2). Nearly all patients were treated with radiotherapy (98.3%, n=59), and most of them had multimodality treatment, including chemotherapy (70%, n=42) and surgery (65%, n=39). A significant proportion of respondents held higher educational degrees (43.4%, n=26), with 15% (n=9) currently employed and 10% retired. Detailed demographic information is provided in **Table 1**.

**Table 1:** Patient demographics and clinical characteristics.

Variable	N, (%)
Total number of participants	60, (100)
Patient age (mean ± SD)	52.13 ± 8.52
Spouse age (mean ± SD)	57.05 ± 10.24
Diagnosis	
Cervical cancer	27, (45)
Endometrial cancer	19, (31.7)
Vulvar cancer	12, (20)
Others	2, (3.3)
Treatment received	
Radiotherapy	59, (98.3)
Chemotherapy	42, (70)
Surgery	39, (65)
Employment status	
Unemployed	45, (75)
Employed	9, (15)
Retired	6, (10)
Educational level	
Up to high school	34, (56.6)
Beyond high school	26, (43.4)

SD (Standard Deviation).

### Patients’ expectations, beliefs, and experience

Two thirds (N = 40) of patients believe that SA is somewhat or very important (who scored 5 and above), and these patients tend to be younger (49.98 ± 8.441) vs (56.45 ± 7.045) (*p* value = 0.005) and have younger husbands (53.88 ± 9.53) (*p* value = <0.001). Before treatment, they rated the quality of their SA as 6.95 (+/- 3.039) on a scale of 1-10; where 1 is the lowest/worst and 10 is the highest/best scores. However, after treatment, this rating decreased significantly to 3.90 (± 2.839), (*p* value <0.001). Those patients who reported lower quality of SA post-diagnosis also indicated SA was less important to them in general (*p* value = 0.007) on 1–10 scale question. Among the patients, 78.3% (N = 47) reported a change in their sexual activity post-treatment, with only 48.3% (N = 29) of these patients noting that their husbands had observed this change. Patients whose husbands noticed the change were significantly younger (49.62 ± 8.641) compared to those whose husbands did not (54.48 ± 7.822) (p value = 0.026). This observation was more prevalent among patients with vulvar cancer (p value = 0.057), particularly those experiencing vaginal stenosis after treatment (p value = 0.003). However, none of the patients considered stopping treatment to preserve SA (**Table 2**).

**Table 2:** Patients’ expectations, beliefs, and experience.

SA quality and importance	Mean ± SD
Quality of SA before diagnosis (mean ± SD)	6.95 ± 3.039
Quality of SA after treatment (mean ± SD)	3.90 ±-2.839
Importance of SA currently (mean ± SD)	5.08 ± 3.033
Statement	Patients who responded (Yes) N, %
I noticed a change in the quality of SA	47, (78.3)
My husband noticed a change in the quality of SA	29, (48.3)
I thought of stopping treatment to limit its effect on SA	0, (0)
I believe the effect of treatment on SA should be brought up	53, (88.3)
I believe the effect of treatment on SA should be discussed before treatment	52, (86.7)
I believe the effect of treatment on SA should be discussed with the medical team	53, (88.3)
The effect of treatment on SA was discussed with me by the healthcare team	21, (35.0)
I believe that best timing for SE discussion:	
At diagnosis	15 (25)
Before treatment	25 (41.7)
During follow up	24 (40)
I prefer not to discuss SE	8 (13.3)
I believe that husband should attend the discussion	38 (63.3)
I believe that husband presence during discussion will negatively affect the relationship	9 (15)
Side effects reported by patients:	
Vaginal dryness	39 (65)
Vaginal stenosis	38 (63.3)
Skin discoloration	24 (40)
Psychological effects	13 (21.7)
Post-coital bleeding	19 (31.7)
Social effects	8 (13.3)

A significant majority of patients (88.3%) believe that the impact of treatment on sexual activity (SA) and sexual education (SE) should be discussed during clinic visits, particularly by their medical team. However, only 35% (n=21) reported actually having had this discussion. When asked about the optimal timing for such discussions, 41.7% (n=25) felt it should take place before treatment that affects SA begins. Additionally, 63.3% (n=38) believed their husbands should be present during these discussions, though 15% (n=9) expressed concerns that their husband’s presence could negatively impact their relationship (**Table 2**).

**Figure 1:**
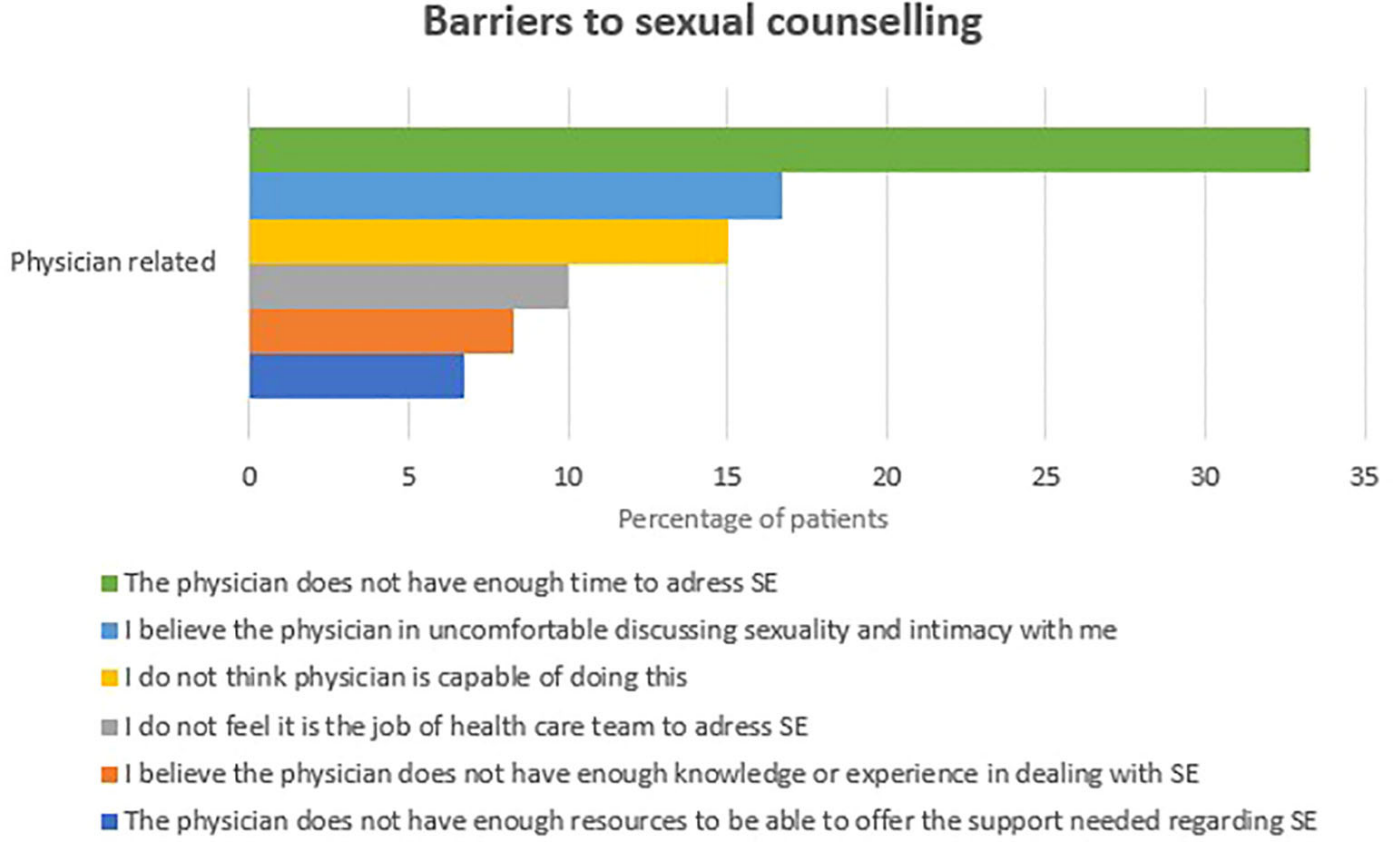
Patient preferences regarding SE at clinic.

Among those who reported having SE discussions at the clinic, 35.1% (n=13) mainly discussed the effects of the tumor and treatments on SA. However, they expressed a desire to address additional topics, such as advice on preserving SA (68.3%, n=41) and understanding the impact of treatment side effects on their husbands (63.3%, n=38). Moreover, many patients showed interest in receiving brochures or written educational materials about SE (61.7%, n=37) or being referred to specialized SE clinics or group therapy (51.7%, n=31) (**Figure 1**).

The most commonly reported side effects after radiation treatment were vaginal dryness (65%, n=39) and vaginal stenosis (63.3%, n=38). However, only 38.9% (n=14) of these patients were willing to use vaginal dilators to address the condition. In terms of quality of life, psychological (34%) and social (10%) effects were the most frequently reported side effects (**Table 2**).

No significant associations were found between patients’ clinical or demographic characteristics and their belief in the importance of discussing sexual education (SE). However, healthcare providers were more likely to discuss SE with patients whose husbands were younger (mean age 53.05 ± 9.795) compared to those with older husbands (mean age 59.26 ± 9.920) (p-value = 0.024), (**Table 3**).

**Table 3:** Associations between demographic characteristics and change in sexual life quality.

Characteristics		Change in sexual life quality		Significance
		Yes	No	
Patient Age	Mean, SD	49.62, 8.64 N= 29	54.48, 7.82 N= 31	0.026
Partner Age	Mean, SD	54.52, 9.94 N= 29	59.50, 10.09 N= 31	0.061
Partner noticed change in sexual life	Yes	29	0	<0.001
	No	18	13	
Type of cancer	Vulvar cancer	10	2	0.057
	Endometrial cancer	7	12	
	Cervical cancer	11	16	
	Other	1	1	
Vaginal dryness	Yes	23	16	0.066
	No	6	15	
Vagina stenosis	Yes	24	14	0.009
	No	5	17	

### Barriers to sexual counseling

**Figure 2:**
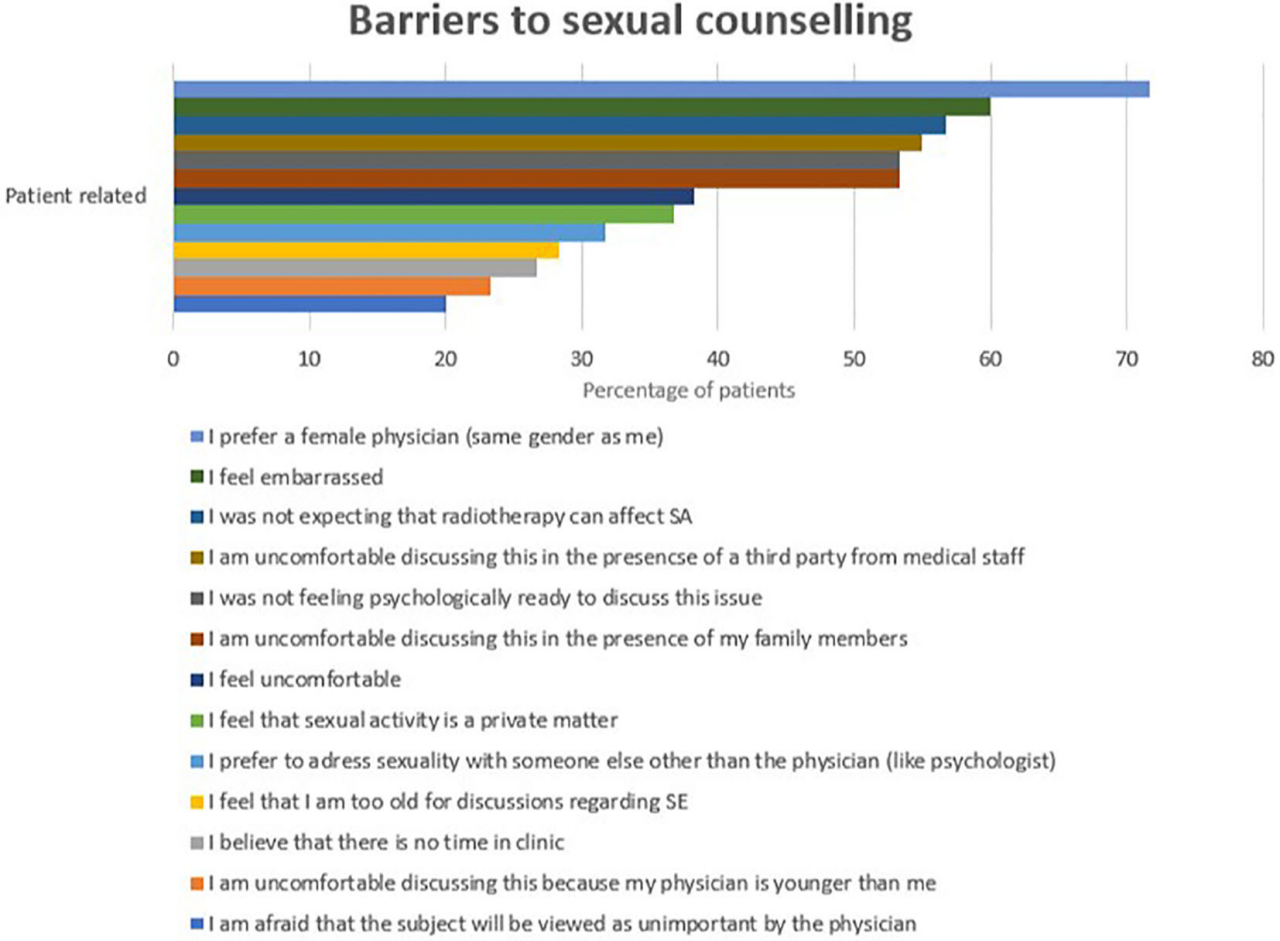
Patient-related barriers into sexual counseling.

Barriers to discussing sexual activity (SA) were categorized into physician-related and patient-related factors, as reported by patients. Among physician-related barriers, the most common were the belief that the physician did not have enough time to address sexual education (SE) (33%, n=20), discomfort with discussing SE (16.7%, n=10), and perceptions of the physician lacking capability (15%, n=9) or adequate knowledge and experience in dealing with SE (8.3%). Additional barriers included patients feeling that discussing SE was outside the physician’s job description (10%) or that the physician lacked the necessary resources to support them (6.7%) (**Figure 2**).

**Figure 3:**
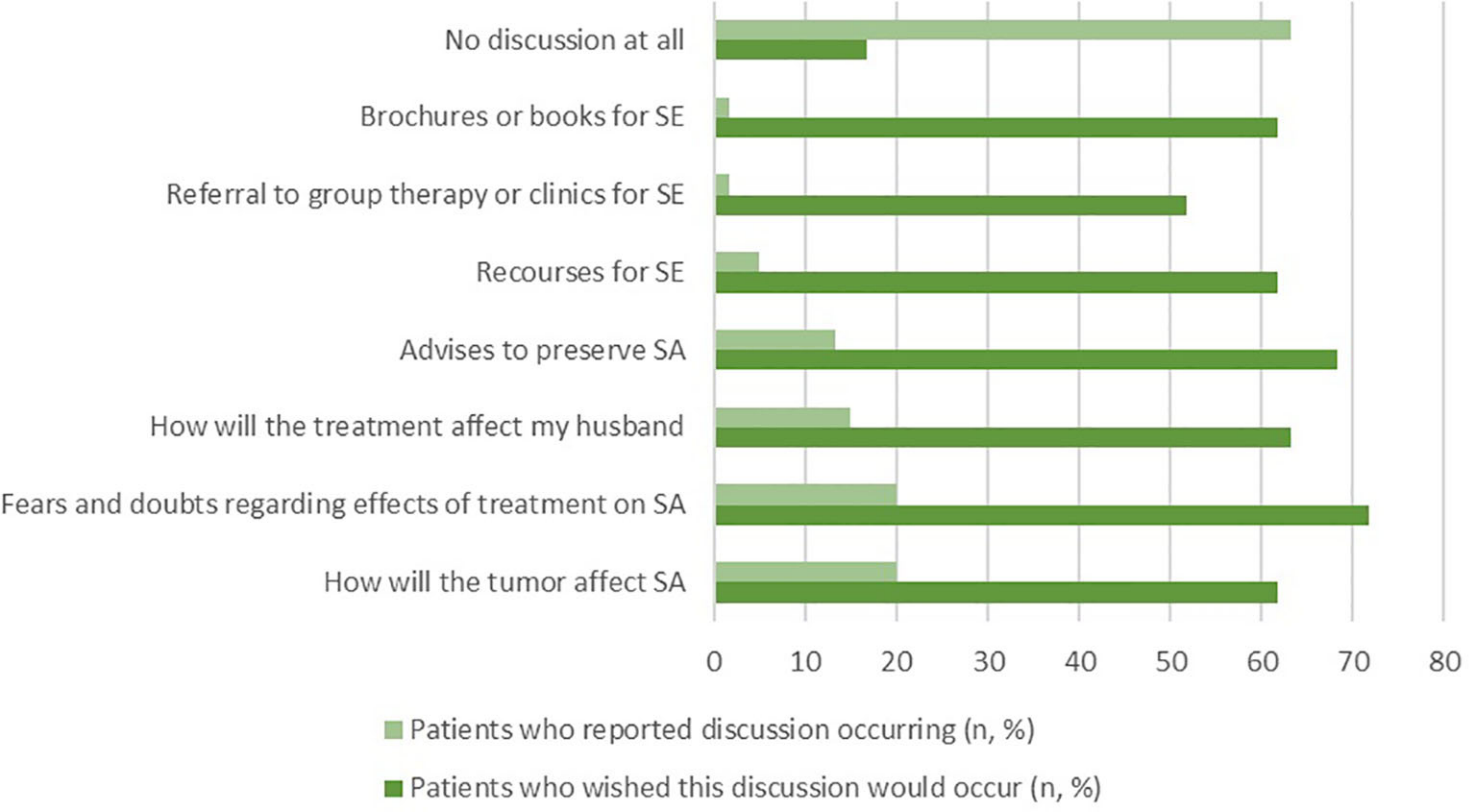
Physician-related barriers into sexual counseling.

On the other hand, the most frequently reported patient-related barriers included a preference for gender concordance, with 71.7% (n=43) specifically preferring a female physician. Other common barriers included feelings of embarrassment (60%, n=36) and a lack of awareness that radiotherapy could affect SA (56.7%, n=34). Patients also cited concerns about a lack of privacy in the clinic due to the presence of third parties such as healthcare staff (55%, n=33) or family members (53.3%, n=32), psychological readiness to discuss SE (53.3%, n=32), and discomfort with the discussion (38.3%). Additionally, some patients considered SA a private matter (36.7%), preferred to discuss SE with someone other than their physician (31.7%), felt their age was inappropriate for discussing SE (28.3%), and noted a lack of time during clinic visits (26.7%). Discomfort related to the physician’s age (23.3%) and concerns that SE might be deemed unimportant by the physician (20%) were also reported (**Figure 3**).

## Discussion

Although, SA among gynecological cancer survivors was addressed before in research from the MENA region (19), this is the first study to address SE among these patients in a cohort from a comprehensive cancer center in the region. Our findings indicate that 88.3% of patients prefer to include SE and discussions about these issues as part of their healthcare.

Although SA appears to be important to at least 66% of our patients, and sexual functions after radiation treatment is usually significantly affected (20), there is a notable gap in addressing SE in clinical settings (3) which is confirmed by our study as only 35% of our patients reported occurrence of SE discussion. On the other hand, 16.7% of our patients preferred not to discuss SA in the clinic. So to ensure high-quality care for our patients, it is crucial to identify those who wish to discuss SE and to address any obstacles or barriers hindering these discussions.

Casey et al. reported that physicians use age of patients as indicator to provide SE, and they suggested to use relationship status and SA as indicators too (7). In our analysis, we found that healthcare providers were more likely to discuss sexual education (SE) when the patient’s husband was younger. This may reflect cultural norms, as sexual activity is typically restricted to marital relationships, accordingly addressing such a topic in unmarried females is considered stigma in our society. In addition this was IRB prerequisite to approve the study. Our study did not identify any specific clinical or demographic characteristics as reliable indicators for providing SE, which may be due to the limited sample size. Given that 88.3% of our patients expressed a preference for discussing SE, we recommend that SE discussions be offered to all patients.

Many of our patients (41.7%) prefers to have SE discussion before treatment that would affect SA or during follow up (40%), This is in contrast with literature, where patients usually prefer discussion during follow up, as they believe that SE discussion at time of diagnosis and treatment will be overwhelming (7). Additionally, 88.3% of our patients prefer that this discussion be led by their healthcare provider, and about half expressed interest in having access to sexual health clinics or group therapy for SE and follow-up. The most common barrier to these discussions was having a physician of male gender, which emphasizing the cultural sensitivity of this topic.

Janelle N. et al. reported that 63% of obstetrician/gynecologists at the U.S routinely assessed patients SA, but fewer than 30% assessed others aspects of SA as satisfaction or sexual problems (3). In our analysis we found that the most commonly addressed aspects were related to the tumor effects on SA (20%) and fear and doubts regarding effects of treatment on SA (20%), but patients actually expressed their desire to have a more comprehensive discussion addressing all aspect of SA and SE.

Delishaj D et al. reported that vaginal stenosis, fibrosis, discharge, bleeding, and dryness are the most common side-effects after high-dose-rate radiation treatment (21). Similarly, Barcellini et al. found that vaginal dryness, stenosis, and pain are the most frequently reported side effects post radiation treatment (22). In our analysis, we observed the same trends, with vaginal dryness (65%) and vaginal stenosis (63.3%) being the most commonly reported side effects among our patients. Notably, only 38.9% of patients with vaginal stenosis expressed a willingness to use vaginal dilators. This may be comparable to the 42% adherence rate over 12 months reported by Law et al. (23). Therefore, if our patients were given the opportunity to use vaginal dilators, they may express almost the same adherence rate.

We do not have sufficient date from traditional radiotherapy era regarding sexual side effects (24), but despite implementing of new radiotherapy modalities in gynecological malignancies treatment, as intensity modulated radiotherapy treatment (IMRT), image-guided radiotherapy treatment (IGRT) and 3-dimentional (3-D) brachytherapy treatment, most studies are reporting high prevalence of mild to moderate vaginal morbidity post radiation treatment (24, 25).

This report is limited by the small cohort size, which is probably related to topic sensitivity primarily, in addition to the lower incidence of gynecological malignancies in Jordan compared to other regions. However, to address this limitation, all eligible patients were included at our center, which resembles the only tertiary healthcare facility that serves as the main referral center for gynecological cancer cases in Jordan, given its comprehensive range of specialized treatment options. Also our patients’ sample includes a high degree of heterogeneity over social, educational and economic factors, for example, the highly educated individuals presented (43%) while urban residents presented (65%). While this may reflect the broader characteristics of our society, again a larger sample size would enhance the generalizability of the results. Additionally, our analysis did not include the perspectives of healthcare workers, such as physicians and radiotherapy clinical coordinators, which limits our understanding of the broader context needed for implementing comprehensive care. Addressing this gap will be a key focus of our future research.

Despite its limitations, this study represents an important initial step toward a more proactive approach to sexual activity (SA) and sexual education (SE) for our patients. We recommend establishing specialized sexual healthcare clinics, led by female physicians, to provide SE for gynecological cancer patients. Such clinics would address many of the barriers reported by patients, such as feelings of embarrassment and discomfort. Additionally, we suggest considering group therapy, which has been shown to effectively address sexual dysfunction in gynecological cancer patients and improve their sexual health and quality of life (12). We also recommend to understand the perspective of the healthcare providers so that a holistic solution can be advocated.

## Conclusion

This is the first study in the MENA region to address sexual education (SE) in gynecological cancer patients. Our findings reveal that SE is a priority for approximately two-thirds of patients, regardless of their age or type of gynecological cancer. However, physicians are not currently providing adequate SE. Therefore, further research is needed to explore physicians’ knowledge, attitudes, and practices related to SE to ensure comprehensive and high-quality care for patients.

## Data Availability

The original contributions presented in the study are included in the article/supplementary material. Further inquiries can be directed to the corresponding author.
